# Evaluation and Treatment of Lumbar Spine Extradural Cysts: A Narrative Review

**DOI:** 10.7759/cureus.60604

**Published:** 2024-05-19

**Authors:** Mohammad Badra, Elie Najjar, Hassan Wardani, Youssef Jamaleddine, Elio Daccache, Hady Ezzeddine, Ramzi Moucharafieh

**Affiliations:** 1 Department of Orthopedic Surgery, Faculty of Medicine, Balamand University, Beirut, LBN; 2 Department of Orthopedic Surgery and Traumatology, Clemenceau Medical Center, Johns Hopkins International, Beirut, LBN; 3 Department of Orthopedics, Center for Spinal Studies and Surgery (CSSS) Queen’s Medical Centee, Nottingham University Hospitals, Nottingham, GBR; 4 Department of Orthopedic Surgery, Faculty of Medicine, Lebanese University, Beirut, LBN; 5 Department of Orthopedic Surgery, Lebanese American University Medical Center, Beirut, LBN; 6 Department of Orthopedics and Traumatology, Clemenceau Medical Center, Johns Hopkins International, Beirut, LBN

**Keywords:** spine endoscopy, surgical endoscopy, spine cyst, surgical excision, endoscopic treatment, histological and anatomical classification, ganglion cyst, synovial cyst, extradural cyst, spine

## Abstract

The main objective was to describe the different types and characteristics of lumbar spine extradural cysts and their optimal treatment options with a focus on endoscopic technique. We searched Pubmed, EMBASE, Medline, and Google Scholar for articles published between 1967 and 2020 using the keywords “Spinal Cyst,” “Extradural Cyst,” and “Lumbar Cyst.” The various anatomical and histological types of the extradural cysts with their presentations, etiologies, imaging, and optimal treatment with a focus on endoscopic techniques were reviewed from the articles.

Lumbar spinal cysts are relatively rare pathologies that might cause radicular symptoms similar to lumbar disc herniation. Spinal extradural cysts are classified either histologically based on the cyst lining tissues (synovial cysts or non-synovial, ganglion cysts) or anatomically based on the structure of origin (epidural cysts, ligamentum flavum cysts, discal cysts, post-discectomy pseudocysts, posterior longitudinal ligament cysts, facet cysts). Surgical excision is the recommended treatment of symptomatic cysts with endoscopic techniques being a viable option.

Extradural lumbar cysts can be identified based on their histological structure or depending on their structure of origin. Regardless of their classification, they could all give similar clinical findings, and the optimal treatment would be surgical excision with endoscopic technique being a viable option with a satisfactory outcome.

## Introduction and background

Lumbar spinal cysts are relatively uncommon pathologies that have been associated with pain and neurological dysfunction such as symptomatic nerve root compression, neurogenic claudication, and even cauda equina syndrome. Most of the reports in the literature discussing intraspinal cysts refer mostly to lumbar juxta-facet synovial cysts. However, little is known about other intraspinal cysts and their pathophysiology or treatment options.

Generally, lumbar spinal cysts are divided into two types based on their location either inside the dural sac (intradural cysts) or outside the dural sac (extradural cysts). Spinal intradural cysts are either arachnoid cysts or enterogenous cysts although there are some reports in the literature about spinal extradural arachnoid cysts [1,2].

Spinal extradural cysts are classified either histologically based on the cyst lining tissues (synovial cysts or non-synovial, ganglion cysts) or anatomically, based on the anatomical structure of origin (epidural cysts, ligamentum flavum cysts, discal cysts, post-discectomy pseudocysts, Posterior longitudinal ligament cysts (PLL), facet cysts). This study aims to discuss the various classifications and types of extradural cysts along with their clinical presentation, etiology, imaging, and optimal treatment (Table 1).

**Table 1:** Classification of lumbar spinal cyst.

Lumbar spine cyst	Intradural cyst	Arachnoid cysts	-
		Enterogenous cysts	-
	Extradural cyst	Anatomic origin classification	epidural cysts, ligamentum flavum cysts, discal cysts, post-discectomy pseudocysts, posterior longitudinal ligament (PLL) cysts, facet cysts
		Histologic classification	synovial cysts or non-synovial, ganglion cysts

This article was previously presented as a poster in the British Association of Spine Surgeons (BASS), Belfast, Northern Ireland, in 2022.

## Review

Methods

We searched Pubmed, EMBASE, Medline, and Google Scholar for articles published between 1967 and 2020 using the keywords “Spinal Cyst,” “Extradural Cyst,” and “Lumbar Cyst.” The various anatomical and histological types of the extradural cysts with their presentations, etiologies, imaging, and optimal treatment including endoscopic techniques were reviewed from the articles with the aim of writing a narrative review.

Data extraction

Titles and abstracts obtained from the databases were assessed for description of extradural cysts based on pathology or structure of origin and treatment options including endoscopic techniques. From the studies included, the data extracted were types of extradural cysts, etiology, symptoms, imaging, treatment options, and use of endoscopic techniques.

Results

Synovial Cyst

This is the most common type of lumbar spinal cyst. Synovial cells line the wall of the cyst and are usually filled with serous, clear, or xanthochromic fluid. Facet joint cysts are usually synovial cysts that typically develop due to degenerative change and are usually continuous with the joint space.

Ganglion Cyst

The ganglion cyst is distinguished from the synovial cyst by the lack of synovial lining. They are not connected to the synovial cavity, and they are usually filled with viscous fluid. Fibrous connective tissue is the main composition of the cyst wall. Ganglion cysts can develop from any connective tissue of the spine. They have been associated with interspinous ligament [3-6], ligamentum flavum [7-14], PLL [15-20], and disc [21-27].

Although histologically different, the synovial cysts and ganglion cysts might share the same clinical and radiological presentation. Both terms have been used interchangeably to describe cysts that develop within the spinal canal causing clinical symptoms of neural elements compression. This review article aims to describe the different anatomical types of spinal extradural cysts, their clinical and radiological presentation, and their optimal treatment methods.

Posterior Epidural Cyst

**Figure 1:**
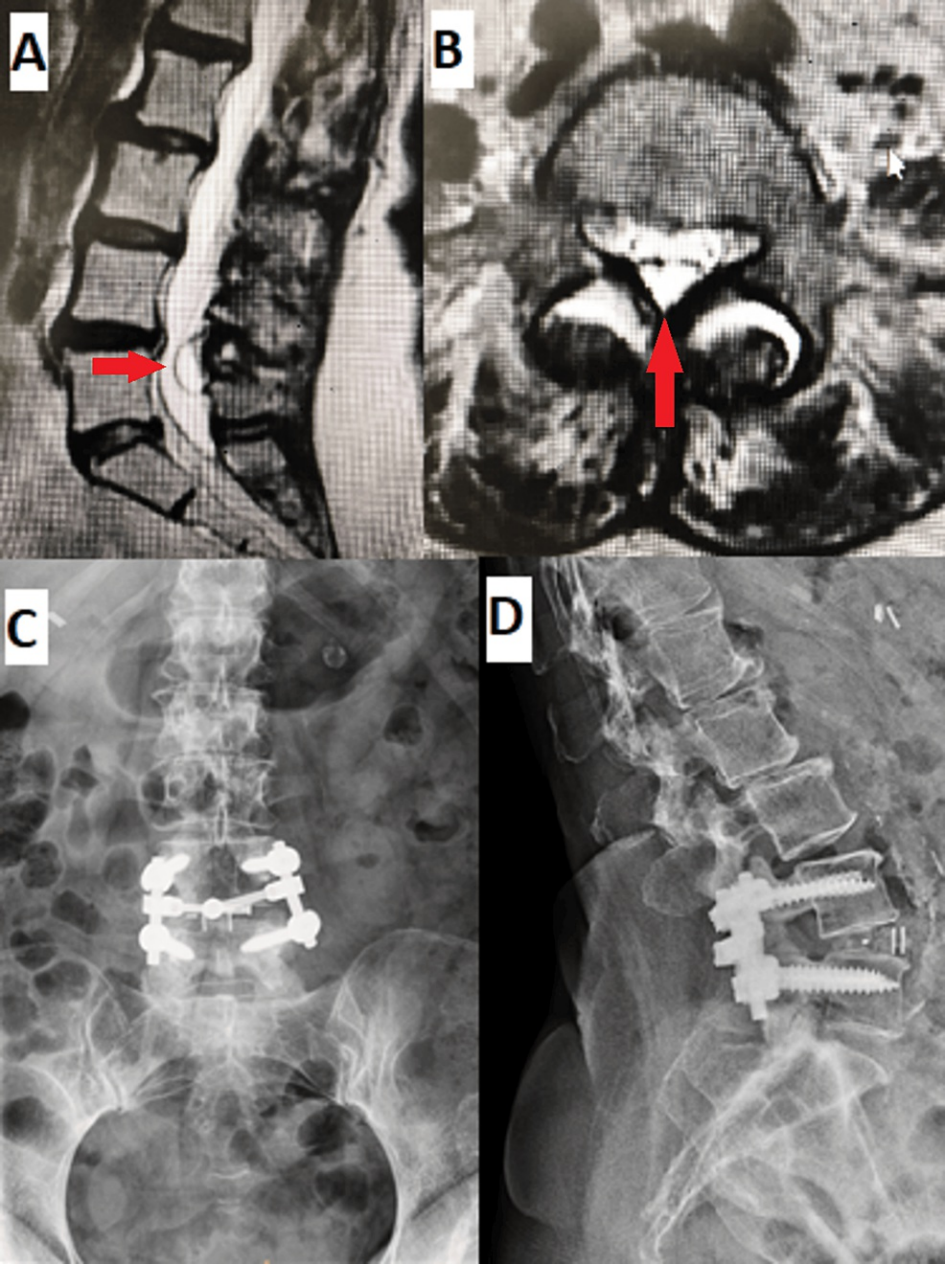
A 63-year-old female with a two-year history of low back pain associated with lower extremity radicular pain secondary to posterior epidural cyst causing neurogenic claudication treated with posterior decompression and fusion. (A) Sagittal MRI and (B) axial MRI showing the epidural cyst (red arrows). (C, D) Postoperative x-rays after posterior decompression and fusion.

Posterior epidural cysts are rare entities that are related to interspinous bursitis and might cause symptoms related to lumbar spinal canal stenosis or sciatic pain secondary to nerve root compression. Degeneration within the interspinous ligament in the lumbar spine, such as occurs in Baastrup’s disease or degenerative spondylolisthesis, can lead to bursitis between lumbar spinous processes in the elderly. Fluid accumulated in the bursa can extend through the midline cleft in the ligamentum flavum and, thus, into the postero-central epidural space and cause posterior compression of the thecal sac leading to spinal canal stenosis (Figures 1A-1D).

Histologically, posterior epidural cysts are typically ganglion cysts with no synovial lining. They are more commonly seen at L4-5, mainly due to the increased mobility at the L4-5 segment. A study conducted using interspinous distraction and stabilizing devices demonstrated that stabilizing the interspinous space leads to cyst resolution, suggesting that cystic degenerative changes are a reaction to the instability caused by the degenerative interspinous ligament that becomes weak and lax [28].

Diagnostic imaging, such as magnetic resonance imaging (MRI), is helpful in the detection of hyperintense signals in the interspinous ligaments, as well as the formation of bursitis in the interspinous space [2]. On MRI, posterior epidural cysts show the same characteristics as the synovial cysts, mainly hyper signal intensity on T2-weighted images, but are localized in the midline. Computed tomography (CT)-guided bursography is also helpful for diagnosis. This method provides not only diagnostic confirmation of the cyst but also allows management through cyst aspiration and steroid injection.

Chen et al. [5] reported on 10 cases of posterior dorsal epidural cysts and noted variable degrees of associated degenerative disc disease, spinal stenosis, and spondylolisthesis with three patients having facet joint effusions. These authors concluded that Baastrup's disease is associated with interspinous fluid and if the fluid bursa is large enough it may extend into the posterior epidural space leading to the formation of posterior epidural cysts and thus causing spinal canal stenosis.

Treatment of posterior epidural cysts is based mainly on the presenting signs and symptoms. If there is only localized lower back pain, conservative treatment in the form of interspinous cyst drainage with an injection of steroids should be tried along with weight reduction and physiotherapy to strengthen the core muscles. Patients presenting with large cysts causing spinal stenosis resulting in radicular symptoms or neurogenic claudication with signs of mechanical instability may need decompression and fusion procedures. If no signs of mechanical instability are present, simple cyst resection to decompress the thecal sac might be a valid option. Kim et al. reported on a full endoscopic treatment of a “spontaneous degenerative epidural cyst” using the interlaminar approach with adequate resection of the lesion and full symptomatic relief [6]. Breed et al. and Baba et al. reported on successful aspiration of the cyst under radiographic guidance with good clinical and radiological short-term outcomes. However, the long-term outcome of such treatment is still unclear [29,30].

Ligamentum Flavum Cyst

These cysts were first reported by Moiel et al. in 1967 [31] and since then many case reports of patients with such pathology have been published [7-14]. Although the pathogenesis is not well understood, it has been suggested that chronic mechanical stress causes ligamentum flavum hypertrophy, myxoid degeneration, necrosis, and consequently cyst formation. Histologic analysis of the cyst usually shows a myxoid degeneration without synovial lining and with clear mucinous fluid.

**Figure 2:**
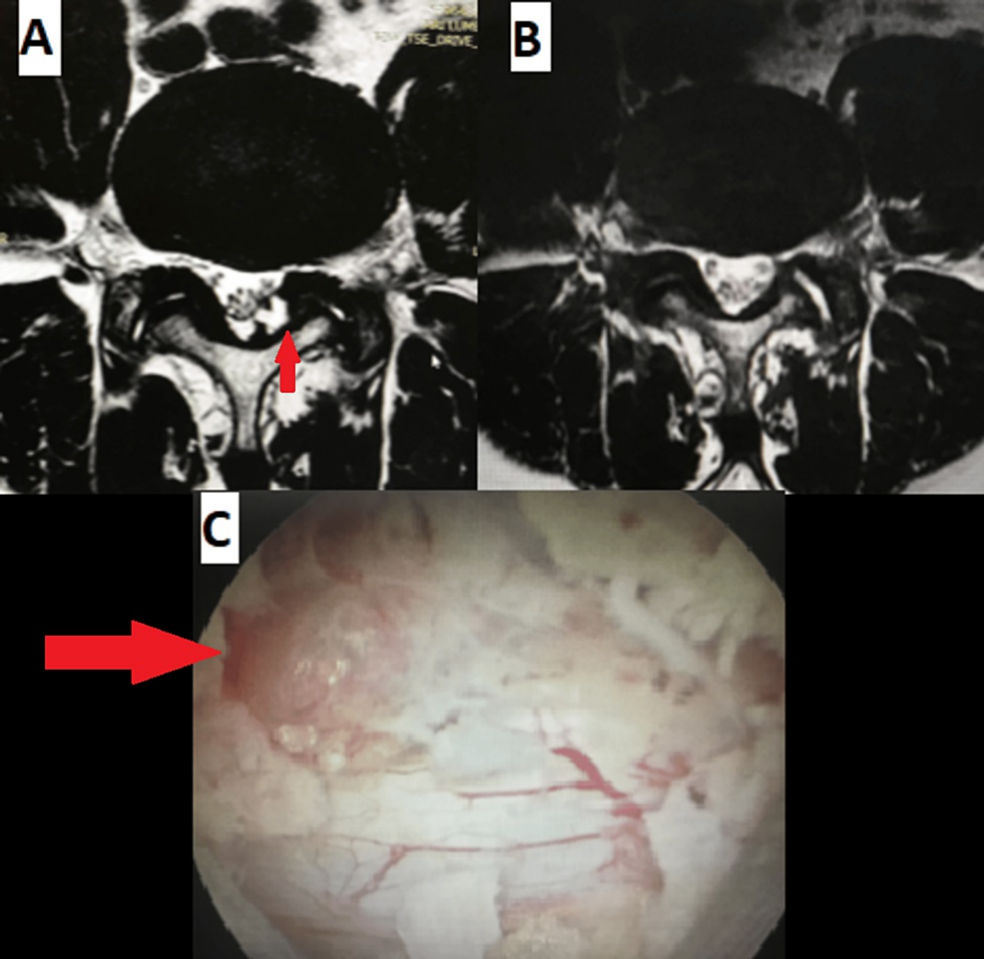
(A) Pre-operative showing ligamentum flavum cyst (red arrow) and (B) Post-operative axial MRI axial images with (C) an intraoperative endoscopic picture showing ligamentum flavum cyst (red arrow) who was treated successfully with full endoscopic interlaminar technique.

Ligamentum flavum cysts are most commonly seen at the level of the lumbar spine but can be seen less commonly at the level of the cervical spine [12,13]. The most common location is the L4-5 level, the most mobile segment of the lumbar spine. On MRI, ligamentum flavum cysts usually demonstrate a high-intensity signal on T2-weighted images and a low-intensity signal on T1-weighted images (Figures 2A-2C).

Patients with ligamentum flavum cysts might be asymptomatic or might present simply with low back pain secondary to degenerative disc or facet disease. Cysts that cause nerve root compression usually present with radiculopathy similar to a herniated disc or lateral recess stenosis.

Treatment of ligamentum flavum cysts is directed mainly at decompression of the neural structures. Conservative therapy has not shown good results and percutaneous aspiration of the cyst is not usually effective. Surgical excision with complete resection of the cyst and the affected ligamentum flavum should be performed to decrease the recurrence rate. Successful endoscopic resection of a ligamentum flavum cyst has been reported by Sharma et al. [14].

Figures 2A-2C show the MRI and the intraoperative images of one of our cases with ligamentum flavum cyst, treated successfully using the full endoscopic interlaminar technique with complete resolution of symptoms. Spinal instrumentation is rarely indicated unless the cyst is associated with signs of segmental instability.

PLL Cyst

**Figure 3:**
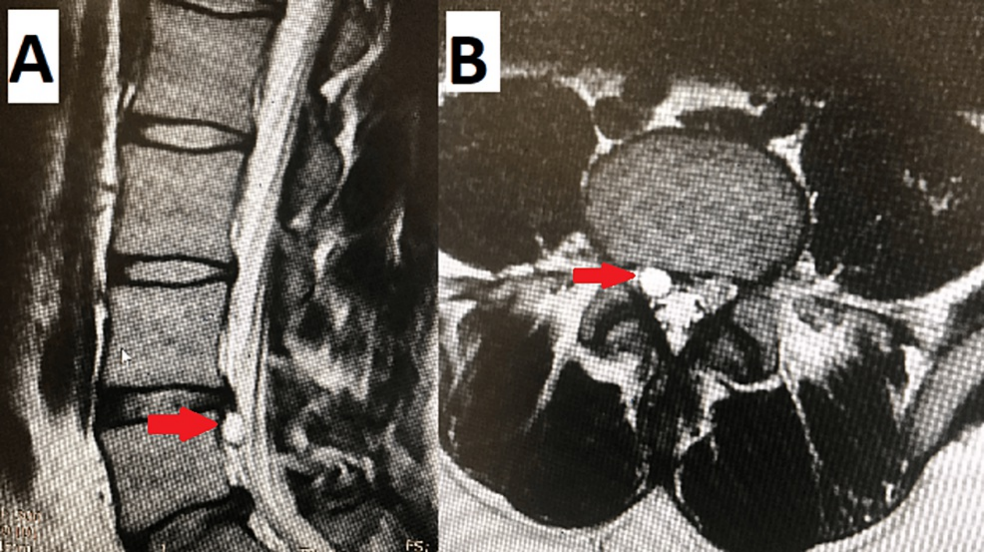
(A) Sagittal and (B) axial MRI with L4/5 posterior longitudinal ligament cyst (red arrow).

Ganglion cyst of the PLL is a very rare pathology. It is usually located posterolateral to the vertebral body, just caudal to the disc space (Figures 3A, 3B). Typically, patients with ganglion cysts of the PLL are young athletic men in their second or third decade of life [19,32]. It has been suggested that injury to a localized area of the PLL secondary to repetitive trauma with subsequent mucous degeneration might be a possible mechanism for the development of PLL cyst [33]. The pressure exerted by a degenerated intervertebral disc on the PLL may cause a small tear in the ligament that subsequently contributes to the development of the ganglion cyst. Other reports suggest that these cysts might arise from the annulus fibrosis of the intervertebral disc itself [20]. PLL cysts may contain serous fluid, mucinous fluid, blood, hemosiderin, and even air [34]. 

On MRI, these lesions are hypointense on T1, and hyperintense on T2, with rim enhancement after intravenous gadolinium administration. PLL cysts can be found incidentally without symptoms, but most commonly patients present with lower back pain associated with radicular pain in the lower extremities. Sometimes hemorrhage within the cyst may dramatically exacerbate existing pain [35].

**Figure 4:**
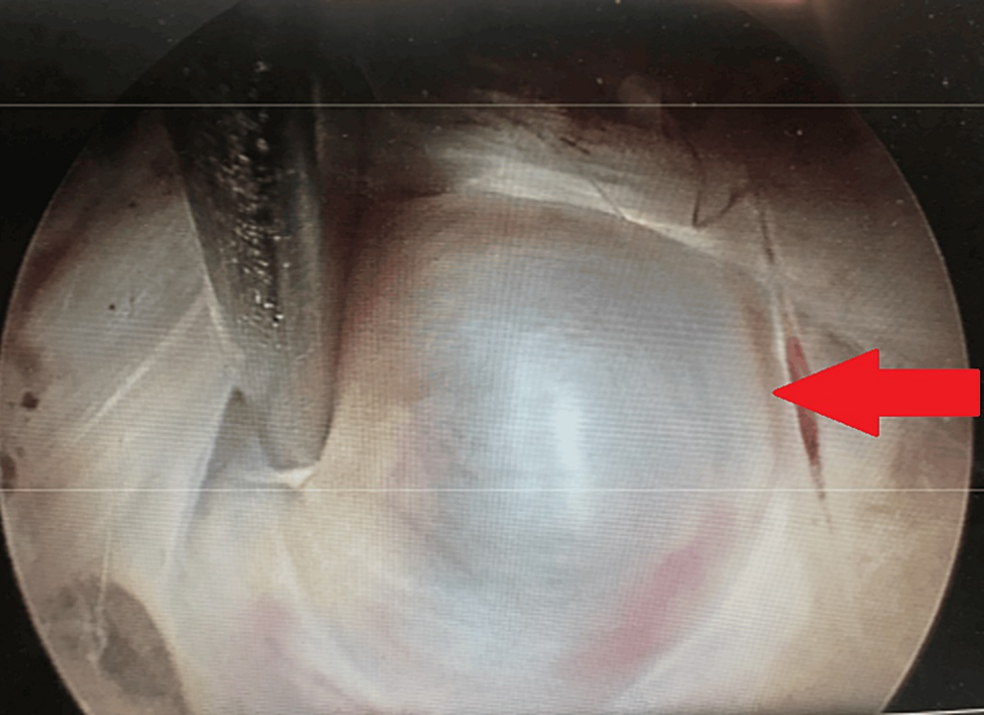
Intraoperative endoscopic picture of L4/5 posterior longitudinal ligament cyst (red arrow).

Intraoperatively, the cyst usually appears as a dark brown cystic mass adherent to PLL and compressing the nerve root (Figure 4). The fluid inside the cyst is hemorrhagic in nature. Histologically, the cyst consists of a thick fibrous capsule with myxoid degeneration. The inner wall of the cyst does not contain synovial lining cells and the fluid demonstrates hemosiderin-laden cells.

Because spontaneous regression is rare, management of a symptomatic ganglion cyst of the PLL is usually surgical. CT-guided needle aspiration of the cyst may provide temporary relief.

The cyst wall should be removed completely to avoid recurrence and should include the removal of a good margin at the cyst base [19]. It is generally believed that a ganglion cyst is likely to recur when the cyst is only partially removed [16]. Surgery can be either done through an open technique or a minimally invasive endoscopic technique.

Discal Cyst

**Figure 5:**
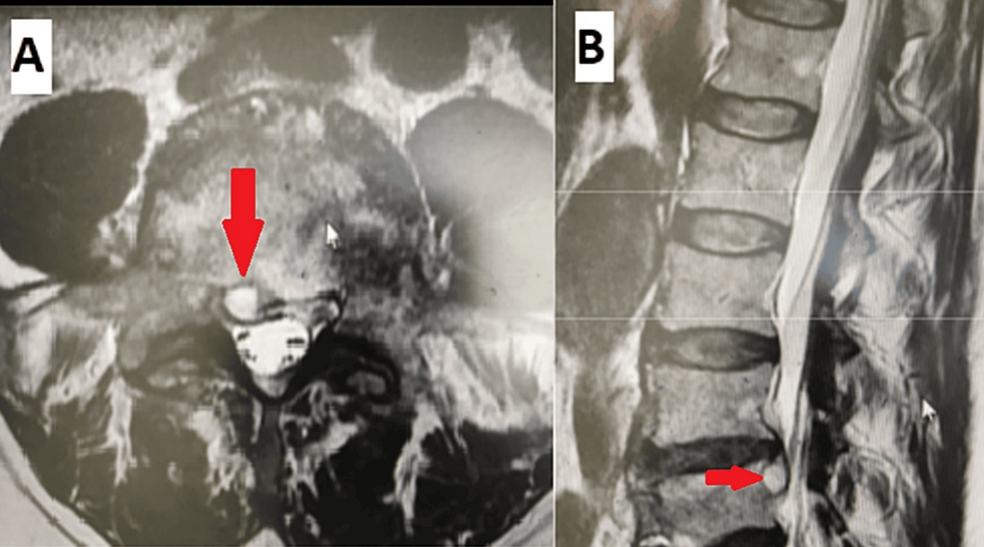
(A) Axial and (B) sagittal MRI of a 55-year-old male patient with right radicular symptoms secondary to discal cyst (red arrow).

These are intraspinal extradural cysts that possess a separate connection to the corresponding intervertebral discs as described by Chiba et al. in 2001 (Figures 5A, 5B) [22]. They are extremely rare pathologies that are most commonly seen in active young male patients [22,24]. The presenting clinical symptoms are similar to that of disc herniation mainly radicular pain secondary to nerve root compression and less commonly low back pain. They are most frequently seen at the L4-5 level, being the most mobile segment in the lumbar spine. Histologically, the cyst wall consists mainly of dense fibrous connective tissue, with no epithelial lining. The cyst content varies from bloody to clear serous fluid.

Several theories have been suggested to explain the pathogenesis of a discal cyst formation. Tokunaga et al. [36] proposed that a discal cyst develops from the absorption of an intervertebral disc herniation. Kono et al. [37] suggested that a discal cyst results from focal degeneration of a herniated disc, which results in an inflammatory reaction forming a pseudomembrane. This theory is supported by the fact that a connection between the cyst and the disc is always found in these patients.

**Figure 6:**
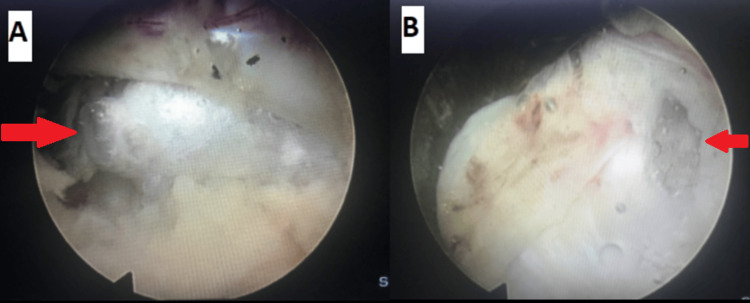
Intraoperative images of a 74-year-old female with the diagnosis of L5-S1 right-sided disc prolapse, found to have a discal cyst, treated with percutaneous endoscopic interlaminar discectomy. (A) Intraoperative endoscopic images of L5S1 right sided disc prolapse with discal cyst discovered (red arrow), and (B) after starting to removing the disc herniation and the cyst with layer opening (red arrow).

Bansil et al. [21] reported on a patient suffering from a herniated lumbar disc, with subsequent transformation into a discal cyst within six months. Kim et al. [26] reported on two cases defining the evolution of spinal discal cysts under percutaneous endoscopy. The pathological components seen intraoperatively, including the pseudocapsule, a hard outer layer, and a tender inner layer, support its origin from a herniated disc. Figures 6A, 6B show the intra-op images of a 74-year-old female patient who was diagnosed with an L5-S1 right-sided disc prolapse treated with percutaneous endoscopic interlaminar discectomy. Discovering a discal cyst with a hard outer layer filled with clear serous fluid is consistent with the theory of Kim et al.

Chiba et al. [22] proposed the epidural hematoma theory. They theorized that a discal injury or disc herniation causing bleeding from the epidural venous plexus results in the formation of epidural hematoma. Hematoma resorption is responsible for the cyst formation. This theory is supported by the fact that most of the cysts contain either hemorrhagic fluid or hemosiderin. Toyama et al. [38] also hypothesized that epidural hematoma is initially formed by rupture of the epidural vein by mechanical irritation from a herniated disc.

The MRI features of discal cysts are similar to those of PLL cysts. They both appear hypointense on T1 and hyperintense on T2‐weighted images with rim enhancement in a contrast study. The principal feature that differentiates disc cysts from PLL cysts is a connecting channel between the cyst and the corresponding intervertebral disc on a discography or a CT discography study. However, it is debatable whether discography is necessary to make a diagnosis as it is an invasive procedure.

Treatment of discal cysts, like any other intraspinal cysts, depends mainly on the presenting signs and symptoms of the patient. Medical treatment modalities include pain control with analgesics, physical therapy, and selective nerve root blocks [27]. Park et al. [39] analyzed 126 patients with lumbar discal cysts and only 19 patients (15%) were successfully treated with conservative treatment.

CT‐guided aspiration of the cyst followed by steroid injection has also been reported [40]; however, there is a high possibility of recurrence after such treatment because the cyst wall or capsule is not removed. It is very important to remove the stalk of the disc as well as the capsule of the discal cyst to minimize the risk of recurrence. Surgical treatment of discal cysts includes open microscopic cyst resection and endoscopic cyst resection. Microscopic resection of the cyst is a simple technique associated with good clinical outcomes and a low rate of recurrence. Park et al. [39] reported that microsurgical resection of discal cysts is an effective surgical treatment with a low recurrence rate.

Endoscopic treatment of discal cysts, whether transforaminal or inter-laminar, has been reported in the literature. Chen et al. [27] reported on nine patients who were treated by percutaneous transforaminal endoscopic surgery with complete symptom resolution and no recurrence. We believe that the interlaminar approach is superior to the transforaminal approach for the endoscopic treatment of discal cysts. When endoscopic surgery is performed to remove a discal cyst, the connection between disc space and cyst capsule should be clearly defined and excised. Incomplete removal of the cyst wall and the base of the cyst with the disc space may lead to a high rate of recurrence. Jha et al. [41] stated that complete removal of the cyst wall through the transforaminal approach is technically difficult as this approach is limited by the bony structures of the intervertebral foramen.

Excision of the corresponding intervertebral disc associated with the cyst is controversial. It is our belief that the removal of the cyst alone is considered sufficient as an effective treatment for lumbar discal cysts with a low risk of recurrence.

Post-discectomy Annular Pseudocyst

PDPs are annular pseudocysts that are seen following disc surgery. They are considered one of the rare causes of recurrent symptoms after lumbar discectomy. This pathology was first described in 2009 by Young et al. [42] who described two patients presenting with recurrent radicular symptoms after microdiscectomy secondary to this pathology.

PDP usually develops over a relatively short period of time after discectomy. Kang et al. [43] reported that “the mean postoperative interval from surgery to PDP detection was 53.7 days.” PDP resembles a discal cyst in the sense it has direct communication with the disc space. However, it is distinguished from a discal cyst by its formation after discectomy.

Symptomatic postoperative lumbar discal pseudocyst seems to have a very low incidence and like discal cysts, young male patients are more susceptible to this pathology. Kang et al. [43] reported an incidence of 1% (15 cases out of 1,503 cases) after endoscopic lumbar discectomy.

Pathogenesis of PDP is unclear, but it has been suggested that inflammatory reaction at the operative site leads to pseudo-membrane, and later pseudo-capsule formation. Kang proposed that physical activity after surgery pumps fluid from the nucleus pulpous through the annular defect into the pseudo-capsule resulting in pseudocyst formation. Young et al. [42] hypothesized a similar mechanism for postoperative discal pseudocyst formation. They suggested that an inflammatory reaction elicited by a herniated disc fragment might form surrounding granulation tissue. If the disc fragment is removed surgically, leaving the pseudocapsule behind, this empty space can allow fluid to accumulate from repetitive loading. Enlargement of this pseudocyst could then cause symptoms similar to a recurrent disc herniation.

Imaging characteristics of PDP are similar to that seen in discal cysts. MRI typically shows a cystic lesion at the site of a previous discectomy with high signal intensity on T2-weighted images, low signal intensity on T1-weighted images, and minimal rim enhancement on post-contrast images. Management of PDP is either conservative or surgical. Conservative treatment consists mainly of medications to treat neuropathic pain related to nerve root compression such as analgesics, nonsteroidal anti-inflammatory drug (NSAID), gabapentin/pregabalin, and physical therapy. Chung et al. [44] retrospectively reviewed 12 symptomatic patients with cystic lesions attached to an operated disc. Of these 12 patients, six were treated conservatively with only analgesics and physical therapy. Near total or complete disappearance of the cysts in an average of 82.7 days (ranging from 23 to 240 days) of initial detection by MRI.

**Figure 7:**
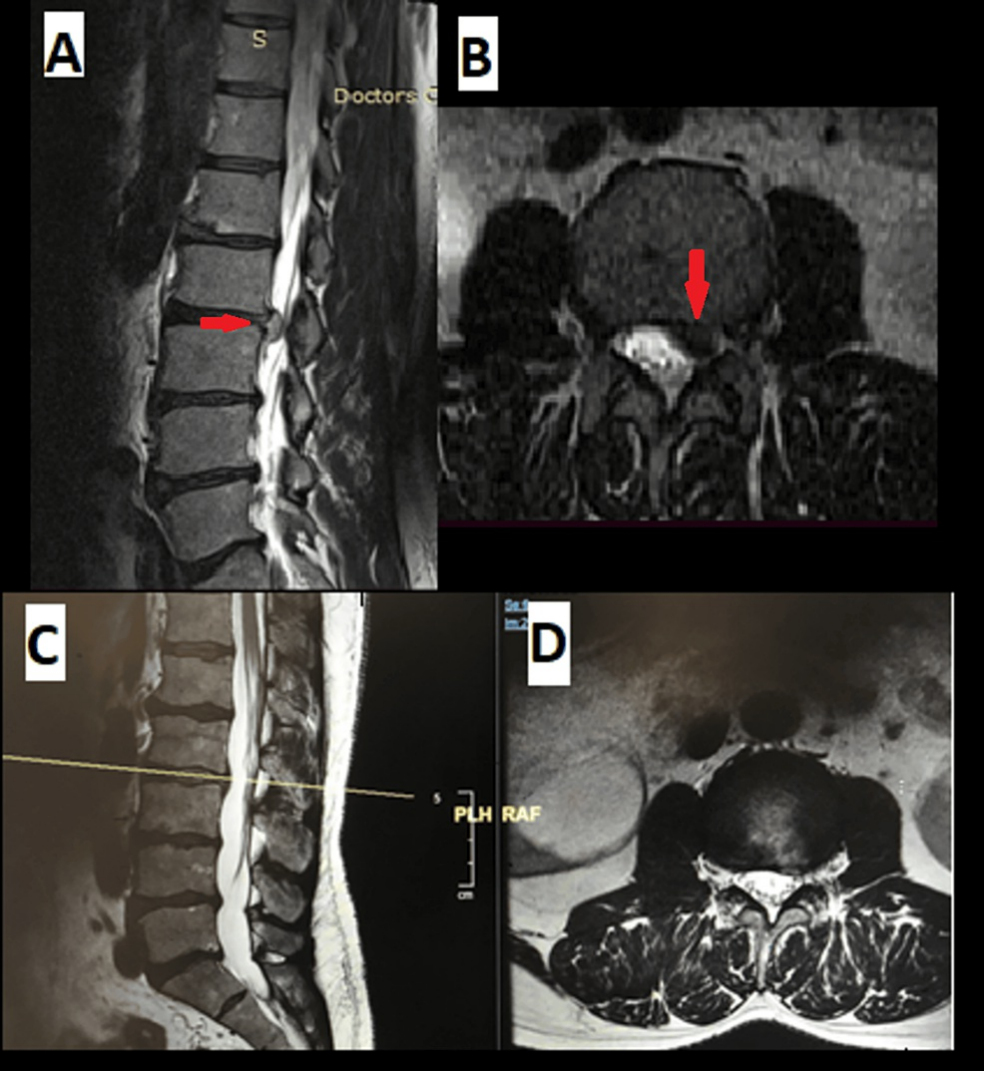
(A) Sagittal and (B) axial MRI of a patient two months post transforaminal endoscopic discectomy for L2-L3 left disc herniation, showing postoperative discal pseudocyst (red arrows). (C, D) Sagittal and axial MRI of the same patient treated conservatively successfully with complete resolution of the symptoms and disappearance of the cyst after three months.

Surgical management can be either through open microscopic or endoscopic techniques. Kang et al. [43] reported no difference in the treatment outcomes between a surgically managed group and a conservatively managed group. In this study, 15 patients with PDP were divided into two groups: surgical treatment group (S) and conservative treatment group (C). Five patients were treated surgically and 10 patients conservatively. In the conservative group, the PDP decreased in size in three cases while one case showed aggravation on follow-up MRI. In the surgical group, one microscopic partial hemilaminectomy and four transforaminal endoscopic discectomies were done with no recurrent PDP. Considering the data, the authors recommended non-surgical treatments initially for patients with PDP, with the possibility of spontaneous regression. Surgical treatment was reserved for patients who failed to respond to conservative treatment. Chung et al. [44] reported on 12 symptomatic patients with PDP. Among the 12 patients, six tolerant patients (group C) were conservatively managed with only analgesics and physical therapy. Of the other six intolerant patients (group S), five were treated surgically and one was treated with needle aspiration under CT guidance with successful pain relief. The authors believed that irrespective of the cyst size, conservative treatment should be tried first, and surgery should be reserved only for those who do not respond to conservative treatment. The outcome appears to be good in the majority of cases with PDP irrespective of treatment type. Figures 7A-7D show a case of PDP that was diagnosed a few months after transforaminal endoscopic discectomy for an L2-3 left-sided disc herniation. The patient presented with a recurrence of his radicular symptoms. He was treated conservatively with analgesics, NSAID, and pregabalin with complete resolution of the symptoms and disappearance of the cyst after three months.

Facet Synovial Cysts

**Figure 8:**
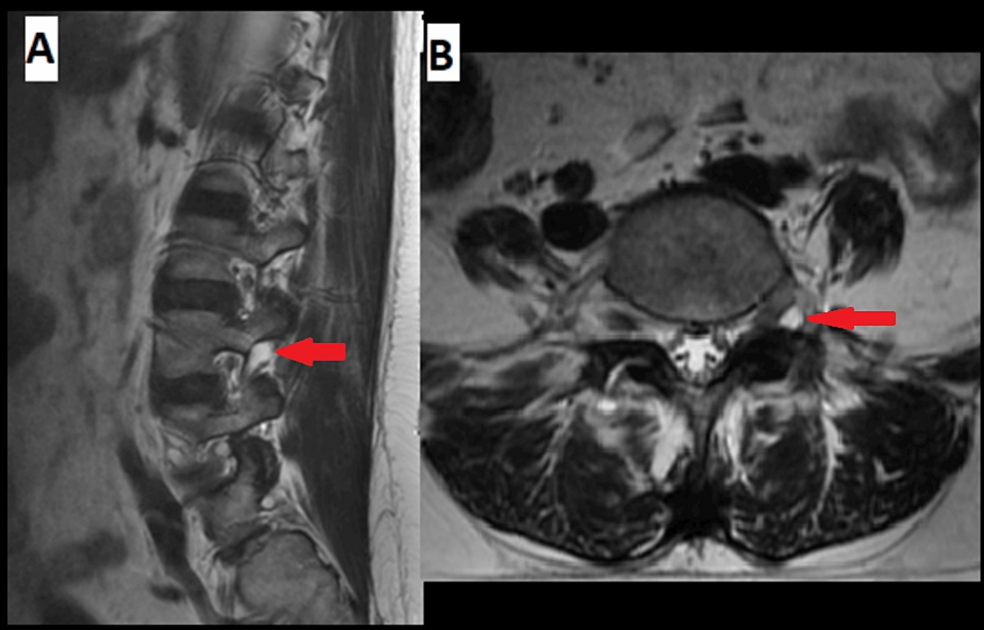
(A) Sagittal and (B) axial MRI showing the facet cyst (red arrow).

Facet synovial cysts arise from the zygapophyseal joints of the spine as a result of degeneration of the facet joint that leads to cystic herniation of the synovial sheath through the facet joint capsule (Figures 8A, 8B). These most commonly occur at the level of the lumbar spine, with the L4-5 being the most commonly involved spine segment. They are often associated with spondylosis and degenerative spondylolisthesis. Spinal synovial cysts are typically found in females during the sixth decade of life [45]. The development of synovial cysts has been linked to a degenerative process that occurs at the level of the spine segment with secondary hypermobility and instability that leads to extensive production of hyaluronic acid by fibroblasts or the myxoid degeneration of collagen tissue in the joint [46].

The wall of synovial cysts consists of a pseudostratified columnar epithelium, and they usually contain clear-colored fluid, in contrast to ganglion cysts which are not lined by synovial epithelium, and are filled with a viscous, gelatin-like fluid. Clinically, facet synovial cysts may be asymptomatic or may present with radicular pain and rarely neurological deficit secondary to nerve root compression. They may also cause neurogenic claudication and cauda equine syndrome. A sudden worsening of symptoms may be indicative of a facet cyst hematoma [47]. MRI is the radiographic modality of choice for the diagnosis of spinal synovial cysts. On MRI scans, these cysts are hypo-intense on T1-weighted images and hyper-intense on T2-weighted images. Peripheral rim enhancement is often observed following gadolinium administration.

Dynamic radiographs may be used to assess instability. It has been shown that not all patients presenting with facet synovial cysts should be labeled as unstable, especially with unilateral cysts. It is important to analyze radiographs to evaluate segmental instability as this may influence treatment options. Treatment of synovial cysts is based on the presenting signs and symptoms. Asymptomatic synovial cysts are usually observed while symptomatic cysts can be treated either conservatively or surgically. Conservative treatment may include bedrest, NSAIDs, analgesics, transforaminal epidural steroid injections, and physiotherapy [45]. CT-guided percutaneous cyst aspiration or rupture has been reported [3,19,28,33,45] to offer symptomatic relief but this is usually only temporary as the rate of recurrence is high due to the fact that the synovial wall of the cyst remains intact allowing regrowth of the lesion.

For synovial cysts associated with neurological symptoms, some forms of surgical intervention should be considered [33,45]. Surgical treatment may consist of simple cyst excision through hemilaminectomy and partial facet joint resection or it may be associated with some form of fusion and instrumentation. The choice of surgical approach depends on several factors, but mainly on the presence of segmental instability, location of the facet cyst (inside the canal, foraminal, and extra-foraminal), and involvement of both facet cysts at the same spinal segment [48]. Treatment of facet joint cysts through minimally invasive spine techniques, either by using tubular retractors or by endoscopy, has lessened the need for fusion in these cases, even in the presence of concomitant spondylolisthesis as these techniques are associated with minimal disruptions of ligamentous structures and bony elements thus decreasing the risk of postoperative segmental instability. Oertel et al. [49] reported on 11 patients operated via an ipsilateral approach for resection of LSC using an endoscopic tubular retractor system. Preoperatively, grade one spondylolisthesis was noted in four patients (36.4%). At follow-up, 10 patients reported no leg pain (90.9%), eight patients reported no back pain (72.7%), and nine patients reported an excellent or a good clinical outcome (81.8%). None of the patients required subsequent fusion procedures. Komp et al. [50] reported on 74 patients with facet synovial cysts who were treated using the full-endoscopic inter-laminar and transforaminal technique. Their results showed that 85% of the patients reported no leg pain or that the pain had been almost completely eliminated, and 11% experienced occasional pain. The complication rate was low.

## Conclusions

Lumbar intraspinal cysts can develop from any connective tissue of the spine. They can cause compression of the neural elements resulting in radicular or claudication-like symptoms. Histologically, degenerative spinal extradural cysts are classified as either synovial cysts or non-synovial ganglion cysts. Description based on the structure of origin is also reported including the interspinous ligament, facet joints, ligamentum flavum, PLLs, and the vertebral disc. The etiology of extradural cysts depends on injuries to the structure of origin. MRI can help differentiate between various cyst types. When symptomatic, surgical excision is recommended with the endoscopic technique described in the literature as a viable option.
